# Food Additives' Impact on Gut Microbiota and Metabolic Syndrome: A Systematic Review

**DOI:** 10.7759/cureus.66822

**Published:** 2024-08-13

**Authors:** Shivani Singh, Oluwatoba T Olayinka, Jaslin Fr, Mah Rukh Nisar, Rudrani Kotha, Sabaa I Saad-Omer, Tuheen Sankar Nath

**Affiliations:** 1 Internal Medicine, California Institute of Behavioral Neurosciences & Psychology, Fairfield, USA; 2 Neurology/Medicine, California Institute of Behavioral Neurosciences & Psychology, Fairfield, USA; 3 Surgical Oncology, Tata Medical Centre, Kolkata, IND

**Keywords:** obesity and diabetes, preservatives, metabolic syndrome (mets), s: gut microbiota, food additives

## Abstract

The human gut microbiota (GM) might play a significant role in the development or remission of metabolic syndrome (MetS) and associated disorders. Contributing factors include diets rich in unhealthy, processed foods that contain preservatives, emulsifiers, and stabilizers. Diet influences the GM's composition, diversity, and species richness in a time-dependent manner. Food additives can alter the GM and contribute to the pathophysiology of MetS by disrupting the intestinal barrier and inducing low-grade systemic inflammation. Our systematic review aims to clarify the relationships among food additives, GM, and MetS. We summarize current knowledge on how food additives interact with GM and the pathogenic role of the microbiota in the development of MetS, including obesity and type 2 diabetes. This review also discusses how disturbances in GM caused by stabilizers and emulsifiers may link to MetS, highlighting the impact of this condition on the development of diabetes and obesity. Furthermore, this review seeks a detailed explanation of how dietary choices related to GM dysbiosis may contribute to MetS. However, more comprehensive and well-designed in vitro, animal, and clinical studies are needed for a better understanding, as research on the role of GM in MetS is still emerging.

## Introduction and background

The term "microbiota" refers to the community of microorganisms, including bacteria, fungi, protozoans, archaea, and viruses, that live in various environments [1]. In humans, the gut microbiota (GM) comprises roughly 30 trillion bacteria, a number comparable to the total human cell count [2]. The main bacterial groups in a healthy adult gut are Firmicutes, Bacteroidetes, and Actinobacteria [3]. These microorganisms have coevolved with humans over millions of years, influencing numerous bodily functions. The GM is now considered a distinct organ due to its extensive role in regulating physiological processes, including energy metabolism. It has a significant influence on metabolic health and can contribute to disorders like obesity and type 2 diabetes (T2D) when its balance is disrupted [4-7]. Dysbiosis, or an imbalance in the GM, is linked to various health issues [8]. Research suggests that this imbalance is a major factor in the development of metabolic syndrome (MetS) and related conditions like obesity and T2D. For example, studies have found that obese individuals with insulin resistance often have a different GM composition compared to healthy individuals, particularly with an increased ratio of firmicutes to Bacteroidetes [9]. MetS includes a group of metabolic disorders such as excessive abdominal fat, high blood pressure, insulin resistance, and abnormal cholesterol levels. These factors significantly raise the risk of developing diabetes and cardiovascular diseases. Both genetic factors and lifestyle choices contribute to MetS, which is often accompanied by inflammation that promotes cardiovascular disease. With obesity rates rising globally, MetS has become a significant health concern, exacerbated by modern technology [10]. Most processed foods contain various additives, like emulsifiers, which improve texture and shelf life, along with preservatives, coloring agents, and flavor enhancers. These additives can negatively impact the GM, leading to dysbiosis, overeating, and MetS. Traditional preservatives like sugar, salt, vinegar, and alcohol are common, but modern processed foods often include compounds like potassium sorbate, sodium benzoate, and calcium propionate. Given that the average adult gets over 60% of their daily calories from ultra-processed foods, it is crucial to understand how food additives affect GM and contribute to MetS. This systematic review aims to clarify the relationships among food additives, GM, and MetS. It summarizes current knowledge on how food additives interact with GM and the role of the microbiota in developing MetS, including obesity and T2D. The review also explores how disturbances in GM caused by additives like emulsifiers and stabilizers may link to MetS and highlights the need for more comprehensive research to better understand these mechanisms.

## Review

Methods

We designed this systematic review and reported results following the Preferred Reporting Items for Systematic Reviews and Meta-Analyses guidelines [11].

The literature search was conducted in PubMed, Scopus, and Web of Science from January 2014 to May 2024 using appropriate keywords and Medical Subject Headings (MeSH) [12-15]. Search terms included "gut microbiota," "metabolic syndrome," "food additives," "artificial sweeteners," "preservatives," and "emulsifiers," combined using Boolean operators (AND, OR). Searches were limited to studies published in English. The full search strategy is provided in Supplementary Table 1. 

**Table 1:** Search strategy

Database	Search Strategy	Key Words	Filters	Search Results
PubMed	(Gastrointestinal Microbiome/immunology" [Majr] OR "Gastrointestinal Microbiome/physiology" [Majr]) or gut flora) AND (("Food Additives/adverse effects" [Majr] OR "Food Additives/analysis" [Majr] OR "Food Additives/poisoning" [Majr] OR "Food Additives/toxicity" [Majr]) or emulsifiers or stabilisers)) (("Disease/etiology" [Majr]) AND ("Metabolic Syndrome/complications" [Majr] OR "Metabolic Syndrome/etiology" [Majr] OR "Metabolic Syndrome/immunology" [Majr] OR "Metabolic Syndrome/microbiology" [Majr] OR "Metabolic Syndrome/prevention and control "[Majr]) or diabetes).	Metabolic syndrome, food additives, gut microbiota, gut flora, metabolic syndrome, diabetes, obesity	Free full text articles in the last 10 years	60.
Google Scholar	Food additives, gut microbiota, metabolic syndrome	Food additives, gut microbiota, metabolic syndrome		5170
Research Gate	Food additives, gut microbiota, metabolic syndrome			100

The final combined MeSH strategy for PubMed is as follows: (("GastrointestinalMicrobiome/immunology" [Majr] OR "Gastrointestinal Microbiome/physiology" [Majr]) OR gut flora) AND (("Food Additives/adverse effects" [Majr] OR "Food Additives/analysis" [Majr] OR "Food Additives/poisoning" [Majr] OR "Food Additives/toxicity" [Majr]) OR emulsifiers OR stabilizers) AND (("Disease/etiology" [Majr]) AND ("Metabolic Syndrome/complications" [Majr] OR "Metabolic Syndrome/etiology" [Majr] OR "Metabolic Syndrome/immunology" [Majr] OR "Metabolic Syndrome/microbiology" [Majr] OR "Metabolic Syndrome/prevention and control" [Majr]) OR diabetes). The search strategy used in different databases is shown in Table 1. 

Keywords for searches in Google Scholar and ResearchGate included food additives, food preservatives, emulsifiers, stabilizers, GM, metabolic syndrome, diabetes, and obesity. These keywords were combined in varying combinations using the boolean operators AND, OR, and NOT.

Inclusion and exclusion criteria

We included systematic reviews, review articles, and literature reviews published in English from 2014 to 2024, focusing on topics relevant to our research, papers related to the subject of study, outcomes of interest, and papers with clear methodology. We excluded animal studies, unpublished or gray literature, publications without a reported methodology, and papers not written in English. The inclusion and exclusion criteria are detailed in Table 2.

**Table 2:** Detailed inclusion and exclusion criteria

Inclusion Criteria	Exclusion Criteria
Systematic review, review articles, literature review	Animal Studies, unpublished or grey literature
Papers from the past 10 years	Papers before last 10 years
Papers in English language	Papers not published in the English language
Papers with an outcome of Interest	Papers with no outcome of interest
Papers related to the subject of study	Papers not related to the subject of study
Papers with clear methodology	Papers with no clear methodology

Analysis of study quality/bias 

We critically evaluated 10 selected studies for quality using standard quality assessment tools; eight studies were deemed medium or high quality and were included in the review. The Scale for the Assessment of Narrative Review Articles checklist was used for traditional reviews. Detailed scores and quality assessments for each study are provided in Table 3. Multiple papers were included to compare the results found in this systematic review [16-47].

**Table 3:** Summary of SANRA checklist for quality analysis

SANRA Checklist	Scoring of Articles Included in Review, Author (Year)							
	Roca-Saavedra et al. (2018) [23]	Lazar et al. (2019) [24]	He et al. (2017) [36]	Erejuwa et al. (2014) [41]	De Siena et al. (2022) [39]	Thomas et al. (2022) [17]	Gálvez-Ontiveros et al. (2020) [34]	Festi et al. (2014) [20]
Justification of the article’s importance for the reader	2	2	2	2	2	2	2	2
Statement of concrete aims or formulation of question	2	2	2	2	2	2	2	2
Description of literature search	1	1	1	2	2	2	2	2
Referencing	2	2	2	2	2	2	2	2
Scientific reasoning	2	2	2	2	2	1	2	2
Appropriate presentation of data	1	1	2	1	2	2	2	2

Data extraction 

Three investigators independently reviewed titles and abstracts to identify potentially relevant studies. These studies were then subjected to a second screening phase for eligibility, as determined by the criteria listed above. 

Results

**Figure 1:**
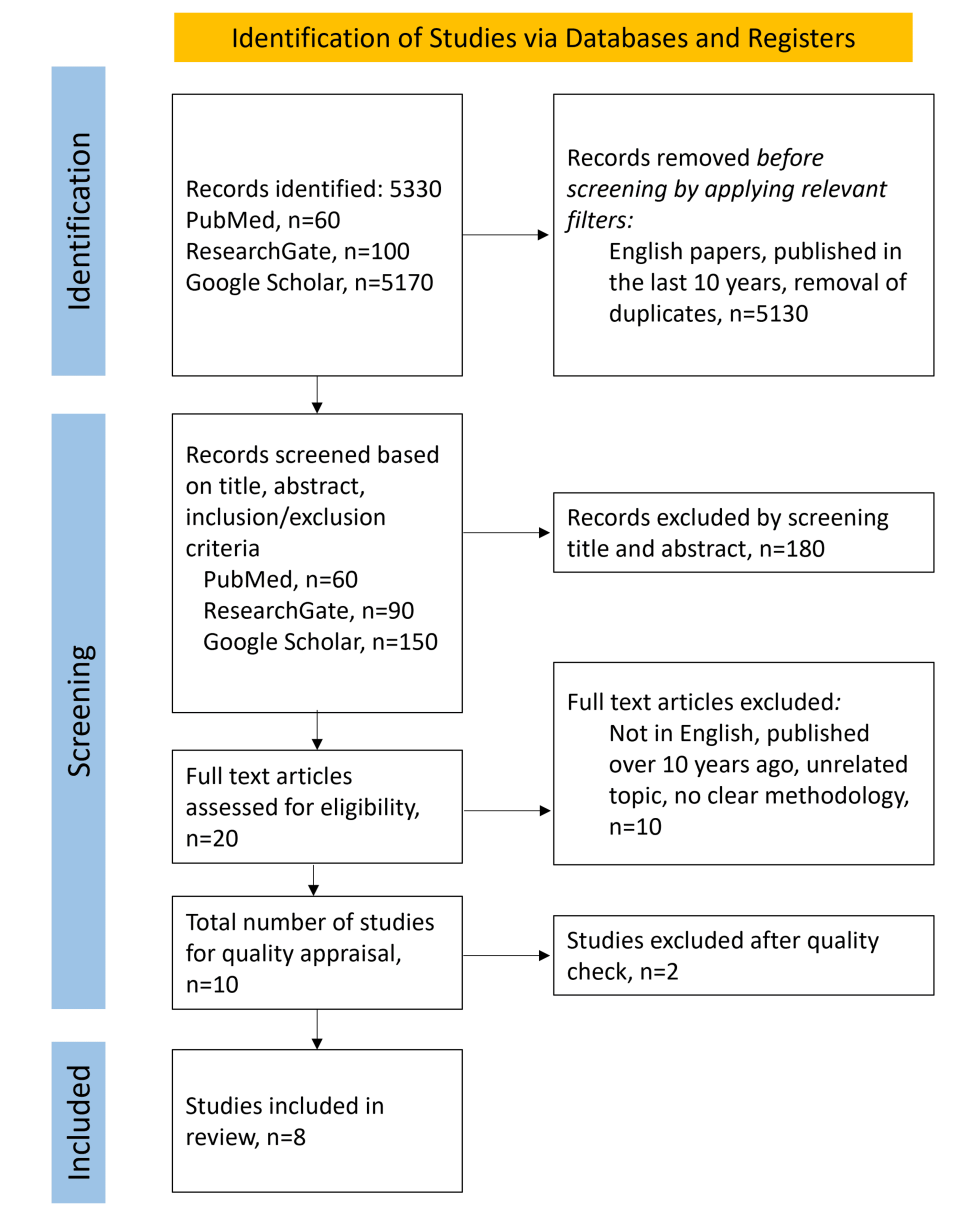
Preferred Reporting Items for Systematic Reviews and Meta-analyses (PRISMA) 2020 flow diagram

Our initial search across three databases using the MeSH strategy, Google Scholar, and ResearchGate identified 5,330 articles. This included 5,170 articles from Google Scholar, 60 from PubMed, and 100 from ResearchGate. We discarded 5,130 articles before screening by applying relevant filters, such as language restrictions to English only, publication within the last 10 years, and removing duplicates. We screened 200 papers based on title, abstract, and inclusion and exclusion criteria, excluding 180 after this screening and applying relevant filters. After a thorough quality and bias assessment of the remaining studies, two studies were excluded after a full article assessment. The reasons for ineligibility were documented. Ultimately, we included eight articles relevant to our research topic in this systematic review. The process is depicted in Figure 1.

Discussion 

Role of Emulsifiers in Gut Inflammation and MetS

The intestinal surface is protected by mucus structures that help isolate most gut bacteria from epithelial cells. Substances that disrupt the interactions between mucus and bacteria may exacerbate conditions associated with gut inflammation. Emulsifiers like carboxymethylcellulose (CMC) and polysorbate-80(P80), detergent-like compounds commonly found in processed foods, may promote bacterial translocation across epithelial barriers. A study conducted on mice demonstrated that even relatively low levels of widely used emulsifiers, such as carboxymethylcellulose and polysorbate-80 (E433), led to low-grade inflammation and the development of obesity/MetS [16]. These findings support the hypothesis that the widespread use of emulsifiers may contribute to the increasing prevalence of obesity, MetS, and other chronic inflammatory diseases [17].

Influence of Diet on GM and Metabolic Disorders

A high intake of simple carbohydrates is associated with MetS and gut dysbiosis. Thomas et al. noted that excessive dietary glucose and fructose intake in mice leads to increased intestinal permeability, metabolic endotoxemia, inflammation, lipid accumulation, steatosis, and obesity [17]. High-fructose corn syrup, a cheaper and sweeter alternative to regular sugar, is abundant in processed foods. Its excessive consumption has been linked to T2D and may alter the species richness of the GM. According to Thomas et al., the GM's composition, diversity, and species richness are dynamically influenced by diet. Cani et al. observed that mice on a high-fat diet for four weeks exhibited obesity and insulin resistance [19,20]. These conditions were associated with pro-inflammatory markers and elevated levels of lipopolysaccharides (LPS), components of the cell membranes of gram-negative bacteria found in the GM. LPS elicits a pro-inflammatory response when bound to TLR4, linking microbiota disturbances with dietary factors and metabolic disorders [21,22].

Diversity of GM and Its Implications

GM varies throughout the gastrointestinal tract and is influenced by several factors. Western diets typically result in a GM dominated by the Bacteroides enterotype, likely responsible for gut inflammation linked to MetS. Roca-Saavedra et al. explored the effects of different microbial phyla, suggesting that a diverse GM could indicate good health [23]. Certain bacteria, such as *Lactobacillus*, *Faecalibacterium prausnitzii*, and *Akkermansia muciniphila*, have been associated with preventing diseases like diabetes and obesity. Roca-Saavedra et al. also discuss how dietary emulsifiers and non-nutritive sweeteners (NNS) like Aspartame, Sucralose, and Xylitol affect GM, stating that NNS can alter GM and worsen glucose intolerance [23]. The study indicates that individuals with diabetes and obesity exhibit significant changes in the quantity and composition of their GM. Elevated levels of *Bacteroides sp.*, *Intestinibacter sp.*, *Escherichia coli*, and *Desulfovibrio sp.* are typical in the microbiota of those with T2D. Moreover, it was found that the microbiota of individuals with type 1 diabetes (T1D) contains higher numbers of "pathobionts," including strains of *Blautia*, *Rikenellaceae*, *Ruminococcus*, and *Streptococcus*, while beneficial bacteria like *Lachnospiraceae* and *Veillonellaceae* are diminished. Additionally, those with T2D and obesity are prone to opportunistic infections such as *Bacteroidaceae* and *E. coli*.

These changes in the GM are linked to systemic inflammation, prolonged bacterial translocation, and increased intestinal permeability, all of which are hallmarks of T2D and obesity. The pathophysiology of these conditions is influenced by dysbiosis in the GM, which is associated with metabolic endotoxemia, insulin resistance, and the release of pro-inflammatory cytokines. The dysregulated composition of the GM, marked by the presence of pathobionts, opportunistic pathogens, and depletion of beneficial bacteria, is implicated in the microbial signature of T2D and obesity. This dysregulation contributes to the onset and progression of these metabolic disorders [24]. 

Role of Emulsifiers and Oxidative Stress in Metabolic Disorders

Lazar et al. explore the cellular mechanisms by which oxidative stress contributes to T2D associated with obesity [24]. The study details how increases in adipose tissue due to obesity lead to heightened inflammation and macrophage infiltration, producing pro-inflammatory cytokines such as tumor necrosis factor-alpha and interleukin-6 (IL-6). This inflammation is accompanied by increased release of free fatty acids and abnormal secretion of adiponectin and leptin, which contribute to chronic hyperglycemia and activate glucose self-oxidation pathways. These pathways produce highly reactive hydroxyl radicals and peroxynitrite, which cause oxidative damage to proteins, lipids, and DNA. Additionally, elevated oxidative stress activates signaling pathways, such as NF-κB and p38 MAPK, linked to insulin resistance and vascular complications in T2D [24]. The study also highlights the unique microbial signatures associated with T1D and T2D, emphasizing the increased presence of "pathobionts" and opportunistic pathogens in the microbiota of those affected by these diseases [25]. Yang et al. discuss how pathobionts from chemically disrupted GM can induce insulin-dependent diabetes in mice [25]. 

Ultra-Processed Foods and Metabolic Syndrome

The risk of developing MetS has been positively correlated with consuming ultra-processed foods. A cross-sectional study of 789 participants aged 40 to 70 demonstrated an independent dose-response relationship between ultra-processed foods and MetS [26]. Ivancovsky-Wajcman et al. showed a positive correlation between an ultra-processed food diet and individual components of MetS, such as hypertension, dyslipidemia (characterized by high triglyceride levels and low high-density lipoprotein levels), and impaired fasting glucose [27].

Dietary Habits and Gut Microbial Communities

Xu et al. reported that individual differences in dietary habits and nutritional status lead to variations in the composition of gut microbial communities along the gastrointestinal tract [28]. Sohail et al. examined how GM contributes to the development of MetS, which, in turn, influences the pathophysiology of diabetes [29]. The review by Kornienko et al. explores the impact of food additives on the human microbiota [30]. They discuss the evolution of the gut microbiome, its physiological roles, and its relationship with disease. They detail common food additives such as titanium dioxide, essential oils, carboxymethylcellulose, sodium sulfite, nisin, potassium sorbate, sodium benzoate, sodium nitrate, Polysorbate-80, and sweeteners like neotame, aspartame, saccharin, sucralose, and acesulfame potassium. Their review describes how these chemicals can alter microbial populations, potentially fostering the growth of harmful bacteria and inhibiting beneficial ones [30].

Impacts of Food Additives on GM

Food additives can significantly affect the human microbiota by promoting the growth of certain bacterial communities while inhibiting others. This alteration can change the body's immune and metabolic pathways. For instance, emulsifiers like carboxymethylcellulose and polysorbate-80 have been shown to increase intestinal permeability and inflammation, potentially contributing to the development of MetS. Kornienko et al. explore the effects of various food additives on the gut microbiome, including artificial sweeteners, preservatives, and titanium dioxide, as well as essential oils, which may influence the diversity of the gut flora and exhibit antibacterial properties [30]. While the direct effects of food additives on the body have been extensively studied, their indirect impacts on the microbiota are less understood.

Zhernakova et al. observed that microbial diversity is negatively impacted by consuming sugar-sweetened sodas [31]. Furthermore, other aspects of a Western-style diet, such as snacking, consuming high-fat whole milk, and increasing overall energy intake, are also associated with reduced microbiota diversity [31]. Maltodextrin, a polysaccharide frequently added to many processed foods, has been linked to changes in the intestinal barrier and may adversely affect GM, potentially exacerbating MetS, as reported by Gerasimidis et al. [32]. Glycerol monostearate (E471), a common preservative in many processed foods, induces body weight gain [33]. Gálvez-Ontiveros et al. have found that exposure to endocrine-disrupting chemicals, such as di(2-ethylhexyl) phthalate (DEHP), can alter the GM's metabolism and immune response [34]. Research suggests that long-term exposure to low levels of DEHP may result in gut dysbiosis characterized by alterations in the composition and function of the GM [34].

Role of GM in Obesity and Diabetes

Lower levels of short-chain fatty acids are found in individuals consuming a Western diet. Modifying gut flora could enhance the body's ability to absorb energy from food, which could contribute to obesity [35]. He et al. describe how probiotics and prebiotics targeting GM can prevent the development of MetS [36]. Prebiotics may reduce low-grade intestinal inflammation linked to MetS, decrease adiposity, improve glycemic control in individuals at risk of T2D, help manage hyperlipidemia through serum lipid modulation, and regulate energy homeostasis, potentially reducing excessive energy intake and obesity [36]. GM influences the endocannabinoid system's lipid and glucose metabolism regulation, presenting new preventive and therapeutic opportunities for MetS.

Insulin resistance and body fat content significantly increased in recipient mice when colonized with GM from conventionally bred obese donors, as demonstrated by Turnbaugh et al. [37]. Most gut bacteria in mice and humans belong to the phyla *Firmicutes* and *Bacteroidetes*. In leptin-deficient obese mice, *Firmicutes* levels increased while *Bacteroidetes* decreased compared to their lean counterparts. Interestingly, human studies have also shown that the composition of GM varies between lean individuals and those who are obese. 

The gut microbiome is also linked to T2D. Research has identified distinct variations in gut flora between individuals with and without diabetes. Diabetes may develop due to the GM's influence on glucose metabolism and insulin resistance. For example, certain microorganisms, such as *Akkermansia muciniphila*, are implicated in regulating or preventing obesity and metabolic diseases [36].

Influence of Food Additives on GM

The food industry extensively uses polysorbates, like polysorbate-80 (E433), as emulsifiers, dispersants, or solubilizers in various products, including bread, cake mix, salad dressing, shortening oil, chocolate, and ice cream. These additives are incorporated into ice creams to enhance their smoothness and melting quality. Research in animals and in vitro has shown that polysorbates can alter the composition of the GM, inducing a pro-inflammatory state [38]. For instance, polysorbate-80 can increase the translocation of bacteria across the intestinal barrier, leading to inflammation. This effect is presumed to stem from increased intestinal permeability, allowing bacterial products like LPS to enter the bloodstream and cause persistent low-grade systemic inflammation and metabolic endotoxemia [39].

Impact of Carrageenans on Gut Health

Carrageenans (E407), a class of food additives used to thicken, stabilize, and emulsify foods, are derived from red seaweed. Carrageenans are a vegan alternative to gelatin in baby formula, common in dairy products, chocolate milk, ice cream, cottage cheese, sour cream, processed meats, and mayonnaise. The potential health implications of carrageenan are concerning, particularly its effects on the intestinal barrier and gut flora. Some studies suggest that carrageenan may disrupt the intercellular connections between intestinal cells, increasing intestinal permeability and leading to a "leaky gut," which can exacerbate inflammation and contribute to MetS [40].

Rising Prevalence of Metabolic Diseases

The incidence of metabolic diseases, such as obesity and diabetes, is increasing, which, in turn, raises the risk of cardiovascular disease. Given the limited efficacy of traditional pharmaceutical treatments, research is exploring alternative approaches, such as modifying the GM [41]. This modification could potentially aid therapy management for various diseases directly (through antimicrobials, dietary adjustments, prebiotics, probiotics, fecal transplants, microbial-derived signaling molecules, or metabolites) or indirectly (via approaches like immunotherapy) [42].

Interaction Between GM and the Immune System

Jin et al. reported that mice lacking the adaptor proteins MyD88 or TRIF (TIR-domain-containing adapter-inducing interferon) exhibited reduced inflammation in adipose tissue and were protected from insulin resistance induced by saturated fats. This finding highlights the interaction between the intestinal microbiota and the innate immune system in causing adipose inflammation [43].

Dietary Shifts and Health Outcomes

Following the industrial revolution, countries with Western lifestyles have experienced a dietary shift from traditional diets to those high in processed foods, fats, sugars, proteins, and various additives, while maintaining low levels of micronutrients and dietary fibers. This is often referred to as the "Western diet." Packaged foods often contain additional ingredients such as colorants, texturizing agents, and preservatives [44]. Dietary fibers are essential for gut health as they promote the growth and activity of beneficial bacteria [45]. Individuals in traditional communities consume approximately 50-120 grams of fiber per day, fostering a more diverse GM indicative of good health.

Benefits of the Mediterranean Diet

The anti-inflammatory effects of a Mediterranean diet enriched with virgin olive oil or nuts were demonstrated through reductions in adhesion molecules, T-lymphocytes, IL-6, chemokines, and monocytes. A Mediterranean diet high in extra-virgin olive oil reduced the risk of T2D by 40% compared to a control diet [47].

Limitations

Our review had several limitations worth considering when interpreting the results. We restricted our review to English-language papers to avoid language bias and excluded potentially relevant research published in other languages. Additionally, we only used freely accessible full-text articles, limiting the total number of studies reviewed.

## Conclusions

The available literature highlights the potential health implications of the interactions among food additives, GM, and the MetS triad. Although promising initial results suggest the efficacy of manipulating GM to treat metabolic diseases, the field is still in its infancy, and comprehensive studies are necessary to establish clear causal relationships and fully exploit this therapeutic potential. A dearth of animal studies and RCTs on this topic indicates a need for more observational studies. While the direct effects of food additives on the body have been extensively studied, their indirect impacts on the microbiota remain less understood and warrant further investigation into the complex metabolic pathways affected and the long-term health consequences. Additionally, more precise assessments of emulsifier intakes and the potential cumulative effects of various food additives are needed. Emulsifiers should be accurately labeled on food products to improve intake estimation. Developing a comprehensive database detailing all manufactured goods with exact food additive concentrations could be beneficial in assessing population exposure. Future lifestyle guidelines for well-being should include advice on building and maintaining a healthy GM through diet and other means.
